# Impact of human cooperation on vaccination behaviors

**DOI:** 10.1016/j.heliyon.2023.e16748

**Published:** 2023-05-27

**Authors:** K.M. Ariful Kabir

**Affiliations:** Department of Mathematics, Bangladesh University of Engineering and Technology, Dhaka, 1000, Bangladesh

**Keywords:** Dynamic vaccination game, Dyadic game, Social dilemma, Cost-benefit

## Abstract

This paper studies a dynamic vaccination game model embedded with vaccine cost-effectiveness and dyadic game during an epidemic, assuming the appearance of cooperation among individuals from an evolutionary perspective. The infection dynamics of the individuals' states follow a modified S/VIS (susceptible/vaccinated-infected-susceptible) dynamics. Initially, we assume that the individuals are unsure about their infection status. Thus, they make decisions regarding their options based on their neighbors' perceptions, the prevalence of the disease, and the characteristics of the available vaccines. We then consider the strategy updating process IBRA (individuals-based risk assessment) concerning an individual's committing vaccination based on a neighbor's decision. In the perspective of social dilemma, it presents the idea of social efficiency deficit to find the gap between social optimum and Nash equilibrium point based on dilemma strength by considering vaccine decision. The cost and cooperative behavior depend on disease severity, neighbor's attitude, and vaccine properties to obtain a reduced-order optimal solution to control infectious diseases. Vaccine factors (efficiency, cost, and benefit) are crucial in changing human vaccine decisions and cooperative behavior. It turns out that, even in the prisoner's dilemma case, where all defection attitude occurs, vaccine uptake (cooperation) increases. Finally, extensive numerical studies were presented that illustrate interesting phenomena and investigate the ultimate extent of the epidemic, vaccination coverage, average social benefits, and the social efficiency deficit concerning optimal strategies and the dynamic vaccine attitudes of individuals.

PACS numbers.

Theory and modeling; computer simulation, 87.15. Aa; Dynamics of evolution, 87.23. Kg.

## 1 Introduction

The impact of human behaviors on participating intervention policies, for example-vaccination, wearing masks, social distancing, etc., is essential to understand and control the spread of contagious diseases [[1], [2], [3], [4], [5], [6]]. A vaccination game model deals with individuals' voluntary exertion of vaccines during an epidemic, assuming that the people change their strategy based on vaccine factors (cost, effectiveness, etc.) and disease severity [[7], [8], [9]]. Many works were published, including when strategy-changing behavior depends on repeated seasons [10,11] or a single season [12,13], and when strategy relies on several updating strategies: individual-based risk assessment (IB-RA), society-based risk assessment (SB-RA), and direct commitment (DC) [10,14]. Several works have been investigated to explore the epidemic control framework on vaccination in which individuals interact with neighborhoods [[15], [16], [17], [18]]. However, the vaccinating behavior of participants relies not only on the vaccine factors (cost-effectiveness), infection risk, vaccine hesitancy, and self-interest but also on the individual's or their neighbor's cooperative behavior [19]. It would be crucial to consider the human cooperative or defective behavior associated with the neighbor's attitude to model any intervention policy for contagious diseases. Therefore, we intend to develop a novel evolutionary game framework for the dynamic vaccination game considering both vaccine cost-effectiveness game and pairwise two players two strategy game, which occur simultaneously on the local time scale.

To study a disease scenario, a mathematical epidemic model was revealed by the extension of classical epidemiological models [20] to multi-compartmental models with different infective group (symptomatic, asymptomatic, hospitalized, isolation, etc) [21,22], control strategies (quarantine, mask-wearing, vaccination, etc.) [23,24], and some others (see Refs. [[25], [26], [27], [28]]). The classical epidemiological models are based on three main compartments, susceptible (S) individuals, infected individuals (I), and recovered individuals (R). The classical model does not take the prevention or control strategy during disease progression. This drawback is compensated by introducing several new compartments such as vaccination, quarantine, treatment, etc. The second assumption concerns the behavioral dynamics of the EGT framework that are supposed to be called the intervention game model [29]. For instance, studies such as SVIR [13], SVIR-UA [30], cyclic SVIS [31], and others [32] have approached epidemiology by incorporating evolutionary game theory into modified versions of the classical SIR model. Inspired by these approaches, we have developed an epidemic vaccination game model that divides individuals into two subgroups: vaccinated and non-vaccinated. In this model, individuals make choices based on factors such as disease incidence, vaccine cost, and vaccine benefits, influencing their preferred options.

In contrast with most of the research discussed above, we consider pairwise games embedded with vaccine cost-benefit games and epidemic dynamics in the current work. We also present the situation where epidemic and behavioral dynamics based on pairwise and cost-benefit games occur in a local time scale (a single season). This gives a new game approach that differs from previous work based on vaccination game with repeated seasons [10,11,26,27]. In the context of a pairwise game, which is also referred to as a two-player and two-strategy game, there are four potential outcomes when two preferences, cooperation and defection, intersect, leading to various payoffs [[33], [34], [35]]. Two individuals receive a reward (R) for mutual cooperation and a punishment (P) for mutual defection, respectively. When a cooperator encounters a defector, the defector achieves the temptation (T). In contrast, the cooperator achieves the sucker's payoff (S). All these four payoffs satisfy the relationship T > R > P > S to generate the popular Prisoners Dilemma (PD) game when together fear and selfishness are present [36,37]. While only greed arises, the Chicken (CH) game arises for T > R > S > P, and Stag Hunt appears when only anxiety occurs for R > T > P > S. To incorporate the concept of universal dilemma strength (DS) [38] into our game model, we introduce a rescaled version. This rescaling is based on two parameters: the gamble-intending dilemma and the risk-averting dilemma. By considering these parameters, we can account for the entire range of dilemmas present in four distinct games: the Prisoner's Dilemma (PD), the Chicken (CH) game, the Stag Hunt (SH), and the Trivial Game (TR) are respectively classified by; $D_{g}^{\prime }>0$ & $D_{r}^{\prime }>0$ , $D_{g}^{\prime }>0$ & *Dr* ′ < 0, $D_{g}^{\prime }<0$ & $D_{r}^{\prime }>0$, and $D_{g}^{\prime }<0$ & $D_{r}^{\prime }<0$ [9,38]. Unlike some previous works [39], here we combine this pairwise game with a vaccine cost-benefit game embedded with disease incidence on EGT. Here, everyone independently selects whether to cooperate or defect. Cooperation means paying a vaccine cost to get maximum benefit on the cost-benefit game and cooperative behavior (get a reward) in a pairwise game. In the context of the information exchange process, the update intention of an individual on another layer is incorporated into the individual-based risk assessment (IB-RA). In our exploration of the concept of social dilemma, we delve deeper into the notion of social efficiency deficit (SED). The SED serves as a metric to quantify the difference in payoffs between the optimal social scenario and the Nash equilibrium, highlighting the extent of inefficiency in achieving the ideal social outcome [40,41].

In this work, our focus is to embed a bridge between the cost-benefit vaccination game and the dyadic game towards exploring the presence of social dilemmas and deficits in epidemiology and evolutionary game theory. We also assess game classes such as PD, CH, SH, and TR as a form of dilemma strength. Here we analyze the situation on a single season called local time scale in which both game and disease occur in the same time frame, which is different from previous work [39]. Afterwards, to understand the impact of embedded game and epidemiology, a range of numerical simulation and graphical analysis are presented.

## 2 Model and method

Here, we have developed a dynamic vaccination-based social learning framework that incorporates both a pairwise game and a vaccine cost-benefit game. This framework aims to capture the frequency at which individuals update their strategies in deciding whether to participate in a vaccine program or risk getting infected during an epidemic. Our approach assumes that individuals base their decision to participate in the vaccine program on factors such as their cooperative behavior and the perceived cost, benefit, and effectiveness of the vaccine in controlling the spread of the epidemic. The preferences of individuals in making these choices are influenced by their surrounding peers, and the process of strategy updates follow an individual-based risk assessment (IB-RA) rule.

**Fig. 1:**
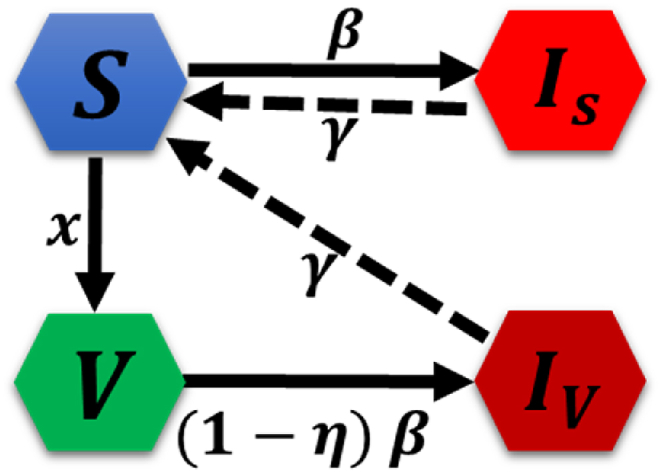
Schematic figure of the model. The population is divided into four states: susceptible (S), vaccinated (V), susceptible infected $\left(I_{s}\right)$, and vaccinated infected $\left(I_{V}\right)$, which pertains in the epidemic season on a local time scale. Here, the parameters x,η,β and γ present for vaccine uptake rate, vaccine effectiveness, disease transmission rate and recovery rate, respectively.

To explore dynamical vaccination with the disease spreading on epidemic dynamics, we assume cyclic epidemic S/VIS model considering four-compartment, susceptible (S), vaccinated (V), susceptible infected $\left(I_{S}\right)$ and vaccinated infected $\left(I_{V}\right)$ at an instantaneous time step (Fig. 1). Here, the total population is supposed to be $S\left(t\right)+V\left(t\right)+I_{S}\left(t\right)+I_{V}\left(t\right)=1$. Unlike the usual epidemic model, the individual will take vaccination at a rate x defined in transition probabilities depending upon two different payoff structures on EGT. In the epidemic process, the imperfect vaccination is conferred, implying vaccine efficiency η, which indicates perfectly immune if the probability is η and invalid vaccination for 1−η. To describe the dynamics of an epidemic in a well-mixed and infinite population, we utilize the compartmental mean-field approximation. This approximation allows us to model the epidemic dynamics using a set of ordinary differential equations:

(1.1) $$\dot{S}=-\beta S\left(I_{S}+I_{V}\right)-xS+{\gamma I}_{S}+{\gamma I}_{V}\text{,}$$

(1.2) $$\dot{V}=xS-\beta \left(1-\eta \right)V\left(I_{S}+I_{V}\right)\text{,}$$

(1.3) $$\dot{I_{S}}=\beta S\left(I_{S}+I_{V}\right)-{\gamma I}_{S}\text{,}$$

(1.4) $$\dot{I_{V}}=\beta \left(1-\eta \right)V\left(I_{S}+I_{V}\right)-{\gamma I}_{V}\text{.}$$

From equations (1.1), (1.4), ***β*** imply the disease transmission rate [per day per person] in which the susceptible or vaccinated people become infected. The fraction of infected people recovered at the recovery rate ***γ*** [per day]. To obtain the reproduction number, let presumed the following two inequalities condition as follows for,

(1.5) $$\left(\dot{I_{S}}+\dot{I_{V}}\right)=\beta \left(I_{s}+I_{V}\right)\left[S+\left(1-\eta \right)V\right]-{\gamma (I}_{s}+I_{v})$$

i) the derivative $\frac{d}{dt}\left(I_{S}+I_{V}\right)<0\text{,}$ if βS+βV(1−η)<γ.
ii) the derivative $\frac{d}{dt}\left(I_{S}+I_{V}\right)>0\text{,}$ if βS+βV(1−η)>γ.

Then, we obtain the effective reproduction number,

(1.6) $$R_{e}=\frac{\beta S+\beta V\left(1-\eta \right)}{\gamma }$$

Here, the existence of stability of the system can be determined by the following proposition. (i) If $R_{e}>1$, then the disease-free equilibrium (DFE) is unstable and (ii) for $R_{e}<1$, the DFE is stable (equations (1.5), (1.6)).

## 3 Evolutionary dynamics

This section assumes three distinct payoff structures with three strategy updating processes to describe human vaccine choice and dilemma in a local time scale (single season) by relying on the vaccination game. The novel point of these payoff structures is the two players' two strategies pairwise game embedded with a traditional vaccination game that contains of a stochastic decision-making aspect. For such games, the choices made by other individuals create both positive and negative incentives, resulting in rewards and punishments; these distinctions are tied to the relative costs and benefits to individuals involved in determining four types of outcomes in the evolutionary game approach.

The two players two strategies pairwise game is a scenario relating to the binary options: cooperator (C) and defector (D) that generated one of the four payoffs: CC, CD, DC, and DD that associated with reward (R), suckers (S), Temptation (T) and punisher (P), respectively. On the other hand, if an individual participates vaccine program with a relative cost of $C_{v}$, the costs are with benefit B. If neither one decides to take a vaccine, there is no cost but get the benefit of free riding. Let the people who participate in the vaccine program and having infected leads to a payoff $C_{v}-1$. Those who failed to keep free riding will pay the payoff −1. Further, in the context of epidemic dynamics, we observe the emergence of four distinct groups among individuals: those who are vaccinated and healthy (HV), vaccinated but infected (IV), successful free riders (SFR) (unvaccinated but healthy), and failed free riders (FFR) (unvaccinated and infected). At each time step, these groups can promptly change their strategies by evaluating their perceived knowledge, influenced by cooperative behavior and the cost-benefit aspects of vaccination. Therefore, the three payoff matrices mentioned above: pairwise game payoffs, cost-benefit payoffs, and the fraction of individuals' payoffs, are given as shown in Table 1, where the labels C and D refer to individuals vaccinated and unvaccinated approach, respectively.

**Table 1:** Payoff structure for the (a) fractions of individuals, (b) vaccine cost-benefit payoff and (c) pairwise game.

	H	I
**V**	V(t) [HV]	$I_{V}\left(t\right)$ [IV]
**NV**	S(t) [SFR]	$I_{S}\left(t\right)$ [FFR]

	H	I
**V**	$B-C_{v}$	$-C_{v}-1$
**NV**	B	−1

	H	I
**V**	1	$-D_{r}^{\prime }$
**NV**	$1+D_{g}^{\prime }$	0

On the allocation of the expected payoff from Table 1, the expected payoff of the cooperator (vaccinated), $x^{+}$ (equation (2.1)), the expected payoff of the defector (unvaccinated), $x^{-}$ (equation (2.2)), and the summation of the product of game, cost, and individuals fraction in the same element termed as average social payoff (ASP) (equation (2.3)) are expressed as follows,

(2.1) $$x^{+}=\left[V\left(t\right)*\left(B-C_{v}\right)+I_{V}\left(t\right)*\left(-D_{r}^{\prime }\right)*\left(-1-C_{v}\right)\right]/\left(V\left(t\right)+I_{V}\left(t\right)\right)$$

(2.2) $$x^{-}=S\left(t\right)*\left(1+D_{g}^{\prime }\right)*B/\left(S\left(t\right)+I_{S}\left(t\right)\right)$$

(2.3) $$ASP=x^{+}+x^{-}$$

## 4 Strategy update process

In the IB-RA, everyone randomly chooses one neighbor to update strategy in a pairwise game by following the Fermi function. We should mention that this update process is like some previous works presented in Refs. [[10], [11], [26], [42]]. However, the setting and perspective are different from previous work because our focus is to embed two different payoffs (pairwise game and cost-benefit game) in the Fermi rule on evolutionary outcomes. Now, if the probability function is $P_{i\leftarrow j}$ for i and j strategies, then the transition probabilities for the eight cases (equations (3.1), (3.2), (3.3), (3.4), (3.5), (3.6), (3.7), (3.8))) are as follows,

(3.1) $$P_{V\leftarrow S}=\frac{1}{1+\exp\left[-\left(1+D_{g}^{\prime }-1\right)/\kappa \right]}∙\frac{1}{1+\exp\left[-\left(0-\left(B-C_{v}\right)\right)/\kappa \right]}$$

(3.2) $$P_{V\leftarrow IS}=\frac{1}{1+\exp\left[-\left(0-1\right)/\kappa \right]}∙\frac{1}{1+\exp\left[-\left(\left(-1\right)-\left(B-C_{v}\right)\right)/\kappa \right]}$$

(3.3) $$P_{IV\leftarrow S}=\frac{1}{1+\exp\left[-\left(1+D_{g}^{\prime }-\left(-D_{r}^{\prime }\right)\right)/\kappa \right]}∙\frac{1}{1+\exp\left[-\left(0-\left(-1-C_{v}\right)\right)/\kappa \right]}$$

(3.4) $$P_{IV\leftarrow IS}=\frac{1}{1+\exp\left[-\left(0-\left(-D_{r}^{\prime }\right)\right)/\kappa \right]}∙\frac{1}{1+\exp\left[-\left(\left(-1\right)-\left(-1-C_{v}\right)\right)/\kappa \right]}$$

(3.5) $$P_{S\leftarrow V}=\frac{1}{1+\exp\left[-\left(1-1+D_{g}^{\prime }\right)/\kappa \right]}∙\frac{1}{1+\exp\left[-\left(\left(B-C_{v}\right)-0\right)/\kappa \right]}$$

(3.6) $$P_{IS\leftarrow V}=\frac{1}{1+\exp\left[-\left(1-0\right)/\kappa \right]}∙\frac{1}{1+\exp\left[-\left(\left(B-C_{v}\right)-\left(-1\right)\right)/\kappa \right]}$$

(3.7) $$P_{S\leftarrow IV}=\frac{1}{1+\exp\left[-\left(\left(-D_{r}^{\prime }\right)-1+D_{g}^{\prime }\right)/\kappa \right]}∙\frac{1}{1+\exp\left[-\left(\left(-1-C_{v}\right)-0\right)/\kappa \right]}$$

(3.8) $$P_{IS\leftarrow IV}=\frac{1}{1+\exp\left[-\left(\left(-D_{r}^{\prime }\right)-0\right)/\kappa \right]}∙\frac{1}{1+\exp\left[-\left(\left(-1-C_{v}\right)-\left(-1\right)\right)/\kappa \right]}$$

The vaccination prevalence will increase or decrease depending on the transition probabilities instantaneously on local time scale (at time t). The mean-field equation is considered for computing the evolutionary mechanism on the social learning process, as follows,

(4) $$\dot{x}=m\left[V\left(t\right)S\left(t\right)\left(P_{S\leftarrow V}-P_{V\leftarrow S}\right)+V\left(t\right)I_{S}\left(t\right)\left(P_{IS\leftarrow V}-P_{V\leftarrow IS}\right)+I_{V}\left(t\right)S\left(t\right)\left(P_{S\leftarrow IV}-P_{IV\leftarrow S}\right)+I_{V}\left(t\right)I_{S}\left(t\right)\left(P_{IS\leftarrow IV}-P_{IV\leftarrow IS}\right)\right]$$

In equation (4), m is the transforming constant that controls the expected vaccine rate from the prospect of altering strategies.

## 5 Result and discussion

We introduce two distinct game structures: a cost-benefit game and a pairwise game. These game structures are implemented within the same evolutionary game model framework, considering an infinite and well-mixed population. By utilizing this framework, we aim to provide a clearer understanding of disease incidence, behavioral considerations, and the presence of social dilemmas. To shed light on these aspects, we analyze key metrics in the form of a phase diagram. Specifically, we examine the final epidemic size (FES), vaccine coverage (VC), average social payoff (ASP), and social efficiency deficit (SED). Through the phase diagram, we aim to provide a visual representation that elucidates the interplay between these metrics, offering insights into the dynamics of the disease, behavioral patterns, and the presence of social dilemmas in the system.

**Fig. 2:**
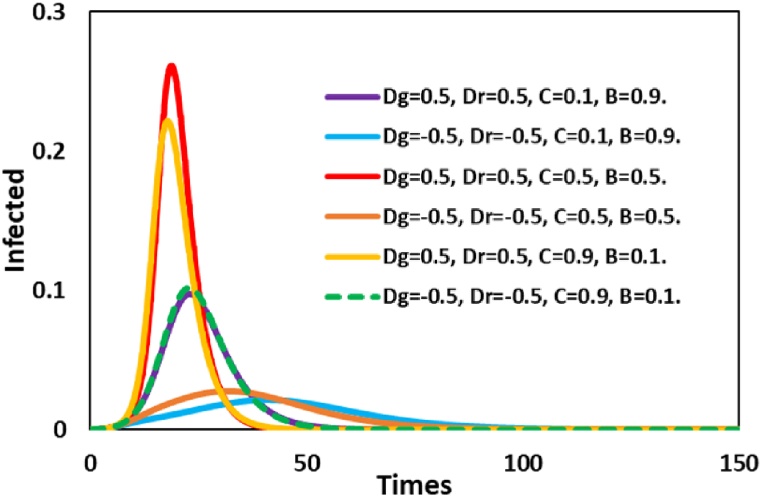
Line graphs for infected individuals for individuals-based risk assessment along with time. Here line graphs present for variation of $D_{g}$, $D_{r}$, vaccine cost (C) and vaccine benefit (B). Others parameters, =0.5,β=1.0, and γ=0.1.

To study the interplay between vaccine cost-benefit and the various game classes in the same framework, we vary vaccine cost, vaccine benefit, and dilemma strength in the system (Fig. 2) along time for infected individuals. It is revealed from the results that a higher infection pick is taking place with intermediate cost and benefit settings for the PD game. Most individuals are not vaccinated due to non-cooperative behavior (for PD game) and intermediate cost-benefit; therefore, the almost full-scale spread of infection arises. On the other hand, for a lower cost and higher benefit, most individuals are taking vaccines resulting in reduced disease. To control the spreading of an epidemic, the fully cooperative game (trivial) and fully defection game (PD) play an essential role in changing the phase between epidemic and control. In the controlling phase, especially for the trivial game $(D_{g}^{\prime }<0$ and $D_{r}^{\prime }<0)$, a fully cooperative society significantly reduces the disease when vaccine cost and benefit favor individuals.

**Fig. 3:**
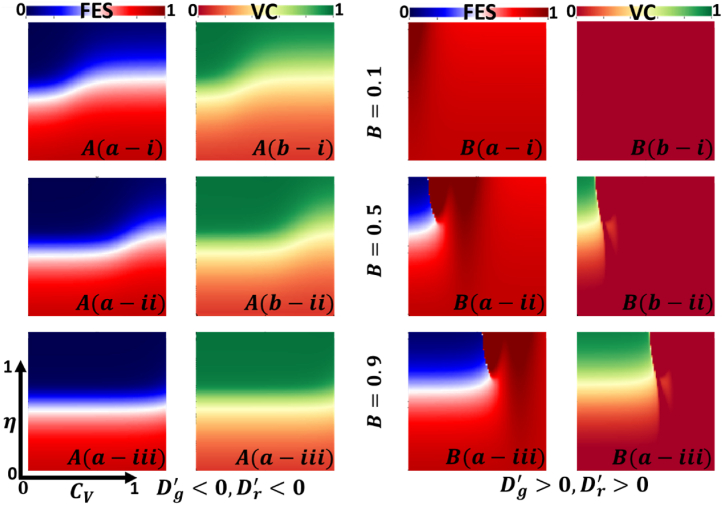
Two-dimensional phase diagram of IB-RA for (a-*) Final epidemic size (b-*) vaccination coverage along vaccine cost and its effectiveness. Panel (A) and Panel (B) present for Trivial and prisoner dilemma game. Also, figure (*-i), (*-ii) and (*-ii) show for vaccine benefit B = 0.1, 0.5 and 0.9. Others parameters, β=1.0, and γ=0.1.

From a holistic perspective, Fig. 3 (A and B) presents the final epidemic size (panel 3 A) and vaccination coverage (panel 3 B) as a 2D heatmap on vaccine cost and vaccine effectiveness for different vaccine benefits (B=0.1,0.5and0.9) and dilemma strength $D_{g}^{\prime }<0,D_{r}^{\prime }<0$ and $D_{g}^{\prime }>0,D_{r}^{\prime }>0)$. With an increase in vaccine effectiveness η, the FES is controlled because the number of vaccinated individuals substantially enhances, as expected. People are most likely to participate in vaccine programs when the vaccine effectiveness is high. Furthermore, with an increase in vaccine cost, FES increases and VC decreases, implying that the lower vaccine price attracts people to participate in the vaccine program. With increased vaccine benefits, most people experience vaccine programs that reduce FES and make costs less sensitive to vaccine decisions depicted in blocks A (a-ii) and A (b-ii). Most individuals concentrating only on higher vaccine benefits make vaccine cost a backseat factor (less sensitive). Consequently, people are most likely to take vaccines when a cooperative society exists; trivial game for $D_{g}^{\prime }<0\text{,}$ and $D_{r}^{\prime }<0$ in Panel A. The benefit of cooperation (vaccination) motivates individuals to participate in vaccine programs, and optimal society follows the *C*-dominant trivial events with reasonably high vaccine benefit, reliability, and affordable price to reduce infection. However, fully unvaccinated area was observed for D-trivial prisoner dilemma game $(D_{g}^{\prime }>0$, $D_{r}^{\prime }>0)$ and less vaccine benefit probability (blocks B (a-i) and B (b-i)). This scenario improves when vaccine benefit to individuals is increased, suggesting that FES and VC outcomes present a bi-stable region for vaccine cost and effectiveness. Block B (a-ii), B (b-ii), B (a-ii), and B (b-ii) present PD in which, above the critical region (red), there is no hope for an individual to get vaccinated due to higher cost. Therefore, it can be generalized that the trivial game performs better to enhance cooperation (vaccination), increase VC, and reduce FES. Interestingly, however, in the region with higher vaccine effectiveness and vaccine benefit with low price, defection is diminished, and fear motivates individuals to cooperate in obtaining the advantages of vaccination. Thus, when vaccine benefit is intermediate, the vaccine cost plays a crucial factor in controlling the disease.

**Fig. 4:**
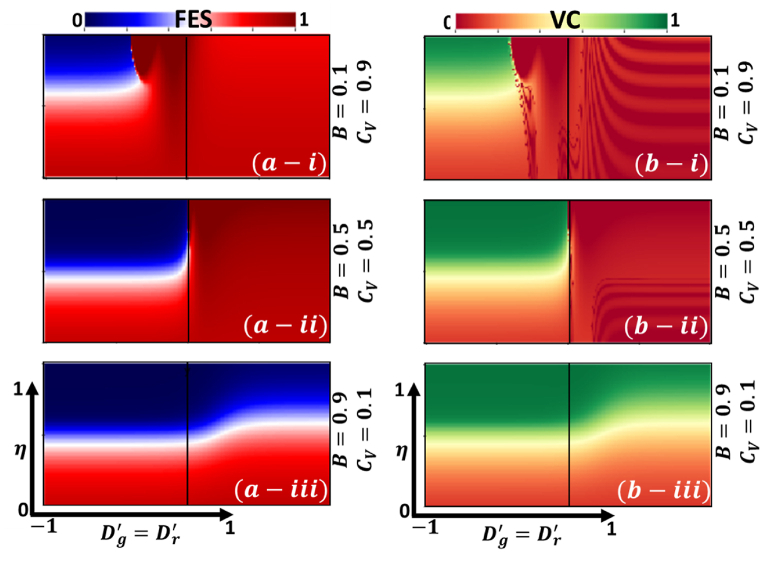
Two-dimensional phase diagram of IB-RA for (a-*) Final epidemic size (b-*) vaccination coverage along vaccine effectiveness (0≤η≤1) and dilemma strength, $\left(-1\leq D_{g}^{\prime }=D_{r}^{\prime }\leq 1\right)$. Sub-panel (a), (b) and (c) present for various settings of vaccine benefit and its cost $\left(B,C_{V}\right)$ as (*-i) (0.1,0.9), (*-ii) (0.5,0.5) and (*-ii) (0.9,0.1). Others parameters, β=1.0, and γ=0.1.

**Fig. 5:**
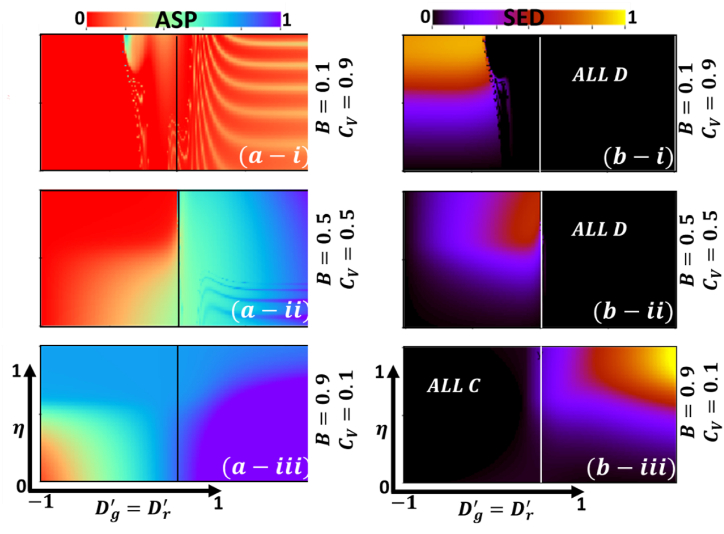
Two-dimensional phase diagram of IB-RA for (a-*) average social payoff (b-*) social efficiency deficit along vaccine effectiveness (0≤η≤1) and dilemma strength, $\left(-1\leq D_{g}^{\prime }=D_{r}^{\prime }\leq 1\right)$. Sub-panel (a), (b) and (c) present for various settings of vaccine benefit and its cost $\left(B,C_{V}\right)$ as (*-i) (0.1,0.9), (*-ii) (0.5,0.5) and (*-ii) (0.9,0.1). Others parameters, β=1.0, and γ=0.1.

Recent works on vaccine attitude and intervention games have revealed that vaccine attitude and corresponding factors rely on vaccine cost and effectiveness. So far, individuals have shown different mindsets concerning reward and punishment in a cooperative game. A critical focus of the present model lies in examining the interplay between self-preference, preferences of others, voluntary vaccination, and their implications on the social dilemma and vaccine deficit within the pairwise game framework. The model explores why certain incentive policies can effectively stimulate vaccination behaviors. To this, we display the 2D heat map (phase diagram) of final epidemic size (FES), vaccination coverage (VC), average social payoff (ASP), and social efficiency deficit (SED) only for IBRA updating rule in Fig. 4, Fig. 5, respectively, along with the vaccine effectiveness (eta) and dilemma strength ($D_{g}^{\prime }=D_{r}^{\prime })$. Meanwhile, the phase portrayed of each block presented for three different vaccine cost-benefit settings, $\left(C_{v},B\right)$ as (*-i) (0.9,0.1) (*-ii) (0.5, 0.5) and (*-ii) (0.1, 0.9), respectively. Here, the game class is categorized based on DS parameters ($D_{g}^{\prime }$ and $D_{r}^{\prime }$) for only Trivial (*C*-dominant) and PD (D-dominant) game classes with game shifting characteristics for variation of different vaccine factors.

Let us start with Fig. 4a and Fig. b for FES and VC. In the case of $D_{g}^{\prime },D_{r}^{\prime }>0$, owing to the higher vaccine cost and lower vaccine benefit to society, the PD region in FES displays a fully infected zone, and VC shows totally unvaccinated. Referring to the knowledge of EGT, such a tendency can be observed in the prisoner dilemma game described as the “D-dominant game.” However, the FES observed in block 4 (a-ii) is not fully D-dominant because low vaccine cost and high vaccine benefits attract individuals to participate in vaccine programs (block 5 (b-ii)). If we suppose that the benefit of vaccine and low price motivates individuals to cooperate, optimal society starts to cooperate from D-dominant to *C*-dominant trivial with relatively moderate vaccine effectiveness to lessen infection. In contrast, the FES in block 4 (a-i) (for lower B and higher $C_{v}$) is not entirely eradicated for Trivial cases $\left(D_{g}^{\prime }<0,D_{r}^{\prime }<0\right)$ because vaccine effectiveness is low, and its benefit and cost are not favorable for individuals to cooperate. Thus, irrespective of Trivial settings $\left(D_{g}^{\prime },D_{r}^{\prime }<0\right)$, D-dominant trivial is observed for higher vaccine cost and DS.

## 6 Conclusion

Recent works of vaccination game model which allows human vaccine behaviors in two processes have different time scales (local and global) and cost-effectiveness game aspect. However, individuals may have distinct mindsets and approaches concerning cooperation and defection attitudes. In this study, we have investigated the embedded two games in a well-mixed population during the epidemic season and explored an effective way to promote cooperation in the context of the vaccination game model. In the previous works [39], the strategy updating process was considered for a global time scale, and both games worked for different situations. Here, we investigate the substantial factor, including social dilemmas, and further study on the same time scale with an embedded game that can promote the cooperation level (vaccination) remarkably. Thus, the variation of vaccine factors (cost, benefit, and effectiveness) on the evolutionary game aspect for dilemma strength allows us to enhance the vaccine uptake of defectors under the action of our presented cooperation promoting mechanism. This modeling approach may be instructive for investigating human behavior during the pandemic and improving cooperation levels (vaccination, mask-wearing, staying home, etc.) in human societies.

## Funding statement

This research did not receive any specific grant from funding agencies in the public, commercial, or not-for-profit sectors.

## Author contribution statement

K M Ariful Kabir: Conceived and designed the experiments; Performed the experiments; Analyzed and interpreted the data; Contributed reagents, materials, analysis tools or data; Wrote the paper.

## Data availability statement

No data was used for the research described in the article.

## Declaration of competing interest

The authors declare no conflict of interest.
